# Speech Driven Gaze in a Face-to-Face Interaction

**DOI:** 10.3389/fnbot.2021.598895

**Published:** 2021-03-04

**Authors:** Ülkü Arslan Aydin, Sinan Kalkan, Cengiz Acartürk

**Affiliations:** ^1^Cognitive Science Department, Middle East Technical University, Ankara, Turkey; ^2^Computer Engineering Department, Middle East Technical University, Ankara, Turkey; ^3^Cyber Security Department, Middle East Technical University, Ankara, Turkey

**Keywords:** face-to-face interaction, gaze analysis, deep learning, speech annotation, multimodal communication

## Abstract

Gaze and language are major pillars in multimodal communication. Gaze is a non-verbal mechanism that conveys crucial social signals in face-to-face conversation. However, compared to language, gaze has been less studied as a communication modality. The purpose of the present study is 2-fold: (i) to investigate gaze direction (i.e., aversion and face gaze) and its relation to speech in a face-to-face interaction; and (ii) to propose a computational model for multimodal communication, which predicts gaze direction using high-level speech features. Twenty-eight pairs of participants participated in data collection. The experimental setting was a mock job interview. The eye movements were recorded for both participants. The speech data were annotated by ISO 24617-2 Standard for Dialogue Act Annotation, as well as manual tags based on previous social gaze studies. A comparative analysis was conducted by Convolutional Neural Network (CNN) models that employed specific architectures, namely, VGGNet and ResNet. The results showed that the frequency and the duration of gaze differ significantly depending on the role of participant. Moreover, the ResNet models achieve higher than 70% accuracy in predicting gaze direction.

## Introduction

Our skills of conversation by means of language, along with the accompanying non-verbal signals, set us apart from other species. Hence, conversation is considered to be one of the important indicators of humanness and human interaction. Recently, Embodied Conversational Agents (ECAs) that allow face-to-face communication are becoming more common. Face-to-face communication implies that interaction should be characterized as an inherently multimodal phenomenon, instead of speech in isolation (e.g., Levinson and Holler, 2014; Kendon, 2015; Mondada, 2016). This is because humans have an ability to send and receive information by means of non-verbal cues such as facial expressions, gestures, gaze, and posture, during a face-to-face conversation. In particular domains, they even correspond to 50–70% of the entire messages that the speaker conveyed (Holler and Beattie, 2003; Gerwing and Allison, 2009).

Gaze is an important non-verbal cue that conveys crucial social signals in face-to-face communication. Although its characteristics depend on individuals and cultural backgrounds, we usually make eye contact with the interlocutor, which, for instance, facilitates joint and shared attention. Even though we have such a tendency, face-to-face conversation is not just an interactive communication where partners constantly sustain eye contact; instead, it involves a sort of transition between gazing toward and away from the communication partner(s). Compared to non-human primates, the specialized morphology of the human eyes, which have a sharp contrast between the white sclera and darker pupil, indicates the special role of revealing gaze direction by the sender and, thus, enables those around the sender to acknowledge about the direction of his gaze. These findings have been well-recognized since the past several decades (e.g., Kobayashi and Kohshima, 1997). We have the ability to make a distinction between directed and averted gaze from a very young age. Even an infant can make such a distinction in the first days of his life (Farroni et al., 2002). The present study focuses on gaze within language context, thus proposing a multimodal approach to computational analysis of face-to-face conversation. In the following section, we present the related work and technical background for the rest of the paper.

### Related Work

#### Gaze in Social Interaction Settings

There exist various functions that the gaze fulfills in social interaction. Expressing emotions is one of the well-known function of gaze (Izard, 1991). An individual should perform eye movements in an appropriate way for the aim of conveying emotional states to an addressee successfully (Fukayama et al., 2002). In addition, gaze takes part in regulation of conversation, transmitting the intention, coordination of turn taking, asserting uncertainty or dissatisfaction, regulation of intimacy, and signaling the dominance and conversational roles (Kendon, 1967; Duncan, 1972; Argyle et al., 1974; Ho et al., 2015).

In recent decades, the development of eye-tracking technologies has enabled robust measurements and novel experimental designs in this field (Gredebäck et al., 2010). However, most of the studies have been performed in laboratory settings by adopting static eye-tracking methods (Pfeiffer et al., 2013), in which participants often monitor the stimulus presented on a computer screen. Although such experimental designs are advantageous in allowing one to provide a controlled procedure, the findings lack generalizability. Eye movements in the field might be different from those in studies conducted with static stimuli in a highly controlled laboratory environment (Risko et al., 2016). This difference can be explained by the two-way function of gaze in social communication. While gaze sends messages about, for instance, floor management or the desire to work together, we also gather information on emotions, intention, or attentional states of others by gazing on them.

Advances in mobile eye-tracking technology have opened the door to researchers who study social interaction in daily-life settings. Broz et al. (2012) studied mutual gaze in a face-to-face conversation with participants wearing mobile eye-tracking devices. They observed a mutual face gaze occurring for about 46% of a conversation. Rogers et al. (2018) also conducted a dual eye-tracking study and reported that the mutual face gaze comprised 60% of the conversation with 2.2 s duration on average.

An important characteristic of gaze in communication is that it is closely connected to speech acts. Accordingly, an analysis of communication in daily settings has to address speech in relation to gaze. In the following section, we introduce systematic approaches to study speech in communication.

#### Speech Annotation

The studies of Natural Language Processing (NLP) involving text mining, automated question answering, and machine translation have gained momentum as a reflection of the developments in Machine Learning (ML) technology (Meyer and Popescu-Belis, 2012; Sharp et al., 2015; Popescu-Belis, 2016). Hence, researchers' attention to discourse analysis has increased in parallel. In the last few decades, a variety of discourse annotation schemas were proposed involving RST (Rhetorical Structure Theory), RST Treebank (Carlson et al., 2001), SDRT, ANN-ODIS, and PDTB (Penn Discourse Treebank) (Prasad et al., 2008). Although there were some common communicative functions in those schemes, there were also inconsistencies between. In order to overcome mapping difficulty between proposed schemes, in the late 1990s, a domain-independent and multi-layered scheme, DAMSL^1^ (Dialogue Act Markup using Several Layers) was proposed. Subsequently, many studies were carried out until the establishment of ISO standard for dialogue act annotation. Eventually, ISO standard 24617-2 “Semantic annotation framework (SemAF)—Part 2: Dialogue acts” was developed (ISO 24617-2, 2012).

The dialogue act is the act that the speaker is performing during a dialogue. In a simplified sense, it is a speech act used in a conversation. A dialogue act has a particular semantic content that specifies the objects, events, and their relations. Furthermore, it maintains a communicative function intended to change the state of mind of an addressee by means of its semantic content. In practice, dialogue act annotation generally depends on the communicative function. A turn represents the duration that the speaker is talking, and it is an important organizational tool in spoken discourse. Turns can be rather long and complex; in this case, they cannot be taken as units to determine communicative functions. They need to be cut into smaller parts called functional segments. Functional segments supply information to determine both the semantic content, namely, “dimensions” (see Table 1), and communicative functions of a dialogue act; for detailed information, see ISO 24617-2 (2012) and Bunt et al. (2017a), and for sample annotations, see DialogBank^2^ (Bunt et al., 2019).

**Table 1:** Dimensions and communicative functions defined in ISO 24617-2.

**Dimension**		**Communicative functions**
Task	Category of dialogue acts that helps to carry out the tasks or activities that inspire the dialogue	General Purpose Functions (GPFs)
Auto-feedback	Category of dialogue acts that take place, in which the sender addresses his processing of past dialogue	AutoPositive, AutoNegative, GPFs
Allo-feedback	Category of dialogue acts that take place, in which the sender argues about the addressee's processing of past dialogue	AlloPositive, AlloNegative, FeedbackElicitation, GPFs
Turn management	Category of dialogue acts that are intended to coordinate the role of the speaker	TurnAccept, TurnAssign, TurnGrab, TurnKeep, TurnRelease, TurnTake, GPFs
Time management	Category of dialogue acts that deal with the allocation of time during the speech	Stalling, Pausing, GPFs
Own communication management	Category of dialogue acts where in the ongoing turn the speaker alters his own speech	SelfCorrection, SelfError, Retraction, GPFs
Partner communication management	Category of dialogue acts where in the ongoing turn the speaker alters the speech of the previous speaker	Completion, CorrectMisspeaking, GPFs
Discourse structuring	Category of dialogue acts that organize the dialogue directly	InteractionStructuring, Opening, GPFs
Social obligations management	Category of dialogue acts carried out to meet social responsibilities such as welcoming, thanking, and apologizing	InitialGreeting, ReturnGreeting, InitialSelfIntroduction, ReturnSelfIntroduction, Apology, AcceptApology, Thanking, AcceptThanking, InitialGoodbye, ReturnGoodbye, GPFs

Dialogue act annotation can be achieved in three main steps: (i) the dialogue is divided into two or more functional segments, (ii) every single functional segment is associated with one or more dialogue acts, and lastly (iii) annotation components are assigned to dialogue acts (see Table 2 for the related components). Although ISO 24617-2 does not provide any specific set for Rhetorical Relations (RRs), for this purpose, it suggests a specific standard, namely, Semantic Relations in discourse, core annotation schema (DR-Core) (ISO 24617-8, 2016).

**Table 2:** Annotation components.

**Component**	**Number**
Dimension	1..1
Communicative function	1..1
Qualifier	0..N
Rhetorical relation*	0..N
**Participant**	
Sender	1..1
Addressee	1..1
Other	0..N
**Dependence relation**	
Feedback**	0..N
Functional*	0..N

*One and only one-dimension, communicative function, sender, and addressee should be attached to a single dialogue act. On the other hand, there might be zero, one, or more qualifiers, rhetorical relations, dependence relations, and participants other than sender and addressee*.

* *Relation is between dialogue acts*.

** *Relation is between either dialogue acts or a dialogue act and a functional segment*.

A multimodal analysis of gaze and speech allows an intuitive understanding of their accompanying role in face-to-face conversation. However, a systematic analysis requires the specification of the relationship between gaze and speech in terms of the identification of specific patterns, which would allow making certain predictions about the interplay of gaze and speech in dialogue. This requires the development of computational models that characterize gaze-speech patterns that emerge during the course of communication. In the following section, we introduce the concept of computational modeling that we employed in the present study.

#### Computational Model

The deep learning approach has greatly improved many artificial intelligence tasks including machine translation, object detection, and speech recognition. In addition to classical AI tasks, researchers have adapted deep learning to various areas. Wang et al. (2017) performed sentiment analysis with data from multiple modalities; Gatys et al. (2016) utilized neural models to produce images in different styles; and Osako et al. (2015) eliminated noise from speech signals.

**Figure 1:**
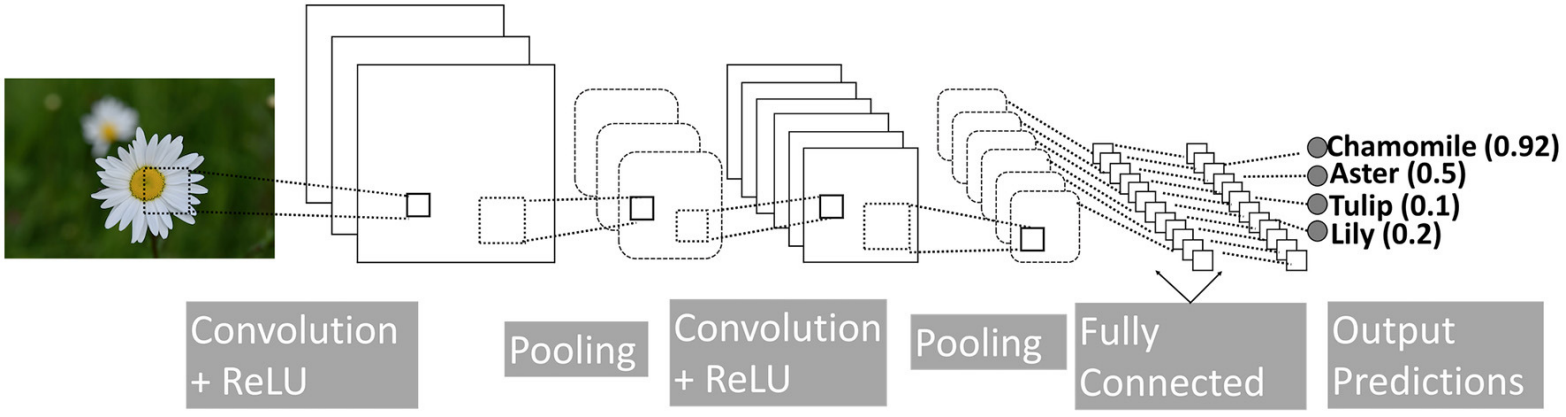
A simple CNN model, composed of convolution, non-linearity (ReLU), pooling, and fully connected layers. The final output is one of the four flowers. Drawing adapted from LeCun et al. (1998).

Convolution Neural Networks (CNNs) are localized versions of fully connected networks (LeCun et al., 1998; Goodfellow et al., 2016). It is based on an important operation, namely, convolution, which integrates the product of two functions. Convolution is useful for calculating change in signals, finding patterns, detecting edges, applying blurs, etc. CNN models that essentially learn the right convolution operations for the task at hand can produce high-accuracy results, especially in the areas of image classification and recognition. A basic CNN architecture includes four fundamental operations: (i) convolution, (ii) non-linearity (e.g., ReLU), (iii) pooling or subsampling, and (iv) classification (Fully Connected), see Figure 1.

Although CNN models are mostly used for image processing, they can be used in the same manner for time series (Fawaz et al., 2019). In this study, we collected the gaze data in the form of a time series and trained 1D CNN networks.

### The Present Study

As reviewed in other articles (e.g., Admoni and Scassellati, 2017; Stefanov et al., 2019), research on the relationship between gaze and speech revealed their close coupling in communication settings (Prasov and Chai, 2008; Qu and Chai, 2009; Andrist et al., 2014). In the present study, we investigated the relation between speech (particularly high-level features of it) and gaze direction (i.e., face gaze or aversion) in a dyadic conversation.

The research into how speech and eye gaze are linked lead to a better understanding of the underlying cognitive mechanisms, but also this relation has been studied for practical applications in Educational Science (e.g., Jarodzka et al., 2017), human robot interaction (e.g., Chidambaram et al., 2012; Ham et al., 2015), web-based conferencing (e.g., Ward et al., 2016), and virtual reality (VR) systems (e.g., Garau et al., 2003; Batrinca et al., 2013). Some of those studies hold under operational assumptions such as simulating gaze aversion through head movements alone, conducting research under highly controlled conditions, which does not reflect real-life settings, or encoding just the presence of human speech rather than exhaustive speech analysis.

The main motivation of the present study is to explore eye gaze and speech relation in a more nuanced and comprehensive manner through employing state-of-the-art technologies and by taking into account the limitations of the previous studies in the field. Moreover, by using the data gathered experimentally, we trained the simplified versions of two deep networks, the ResNets (He et al., 2016) and VGGNet (Simonyan and Zisserman, 2015) that predict gaze direction based on high-level speech features.

Stefanov et al. (2019) showed that listener's gaze direction could be modeled from low-level speech features without considering semantic information, and they concluded that different methods are required for modeling speaker's gaze direction. In successful communication, the listener understands what the speaker says the way the speaker desires. In doing so, the listener takes into account the basic characteristics of the speaker's utterances, as well as the motivation behind the initiation and the history of the dialogue, and even his/her assumptions about the opinions and goals of the interlocutor. We cannot derive the communicative function of a dialogue by considering only the surface form of utterances since the same utterance forms can have different meanings in different conversations between different people. In the present study, to model states of both listening and speaking, we used high-level speech features.

It has been reported that (e.g., Dbabis et al., 2015; Bunt et al., 2017b) as high-level speech features, the dimensions and dialogue acts of ISO 24617-2 standard could be automatically recognized with fairly high accuracy. Therefore, even in case of a fully automated analysis, which can be conducted as a further study, ISO 24617-2 standard is a good candidate for extracting high-level speech features. The analysis of gaze and its ties to co-occurring speech is not a new topic of inquiry (e.g., Ekman, 1979; Zoric et al., 2011; Ho et al., 2015); however, as mentioned above, speech analysis was performed based on syntactic features or just for specific communicative function(s) such as turn taking, instead of adopting comprehensive semantic annotation frameworks. To the best of our knowledge, ISO 24617-2 standard has not been adopted in predicting gaze direction, so far.

In the present study, the speech annotation was handled in two ways: (i) ISO 24617-2 and ISO 24617-8 for annotating discourse and rhetorical relations, respectively, and (ii) an alternative set of speech tags that we proposed based on the roles attributed specifically to the gaze in social communication. The reason of annotating speech with two different methods is to investigate which characteristics of speech will produce better performance in modeling social gaze. In the following section, we present experimental investigation with analysis results.

## Experimental Investigation

### Materials and Design

#### Participants

Twenty-eight pairs involved seven professional interviewers, 4 females (mean age = 33.8, *SD* = 4.72) and 3 males (mean age = 35.7, *SD* = 0.58), with the mean age of 34.6 (*SD* = 3.51); and 28 interviewees, 14 females (mean age = 25.1, *SD* = 2.57), and 14 males (mean age = 25.4, *SD* = 2.68), with the mean age of 25.3 (*SD* = 2.58) took part in the study. Interviewers took part in multiple interviews (*M* = 4, *SD* = 0.93). Participants in each pair did not know each other beforehand. All the participants were native speakers and had a normal or corrected-to-normal vision.

#### Apparatus

Both participants in a pair wore monocular Tobii eye-tracking glasses, which had a sampling rate of 30 Hz with a 56° × 40° recording visual angle capacity for the visual scene. The glasses recorded the video of the scene camera and the sound, in addition to gaze data. Interviewers read the questions and evaluated the interviewee's response on a Wacom PL-1600 15.6 Inch Tablet, which enables users to interact with the screen by using a digital pen.

#### Procedures

The task was a mock job interview. It is adopted from the previous studies (i.e., Andrist et al., 2013, 2014). Eight common job interview questions, adopted from Villani et al. (2012), were translated into Turkish and presented to interviewers beforehand. The interviewer was instructed to ask given questions and also to evaluate the interviewee for each question right after the response. A beeping sound was generated to indicate the beginning of a session. The participants stayed alone in the room throughout the sessions.

### Data and Analysis

Data analysis consists of three main steps. In the first one, we extracted gaze directions of each participant. As the next step, we analyzed audio data for extracting high-level speech features. In the final step, we synchronized gaze direction data with speech annotations.

We have developed an open source application that provides an environment for researchers working in the field without requiring a technical background (Arslan Aydin et al., 2018). It is capable of detecting and tracking conversation partner's face automatically, overlaying gaze data on top of the face video, and incorporating speech through speech tag annotation. It automatically detects whether the extracted raw gaze data is face gaze of an interlocutor or an aversion. In addition, it provides interfaces for speech analysis involving segmentation, synchronization of pair recordings, and annotation of segments. It significantly reduces the time and effort required for manual annotation of eye and audio recording data. Manual annotation is vulnerable to human-related errors, and in addition, automatic annotation with the state-of-the-art methods provide further information that may not be extracted manually such as detecting the coordinates of facial landmarks, taking into account the error margins while annotating the gaze direction or segmentation of the speech at milliseconds precision. The application employs OpenFace (Baltrusaitis et al., 2016) for gaze direction analysis, CMUSphinx^3^ for audio recording analysis, and dlib^4^ for training custom face detector. We generally used interfaces of the developed application in the gaze and the speech tag set analysis.

#### Speech Analysis

Audio stream from each participant's recordings was extracted before performing the speech analysis. The mean duration of the recordings was 09:41.543 (*SD* = 04:05.418) (in mm:ss.ms format). We performed speech analysis with two methods both including segmentation and annotation sub-steps. As the first step of speech tag set analysis, the audio files of sessions were segmented into smaller chunks including sub-words and pauses. The number of segments (*M* = 737.4, *SD* = 414.1) varied depending on the length and the content of the audio. Since the developed application called Sphinx4 libraries for the segmentation of audio files, each segment had a maximum temporal resolution of 10 ms.

Then, in order to determine session intervals and provide synchronization between the pair recordings, we listened to audio segments and identified the ones containing beeping sound. The time offset between the pair's recordings was calculated by using the application interface. Lastly, for improving segmentation quality, the synchronized pair recordings were re-segmented via merging the time interval information of both participants' segments (see resources^5^ for an example and usage of developed application).

At the annotation stage of speech tag set analysis, segments were annotated with the predefined speech labels that we decided to use by benefiting from the founding of previous social gaze studies (e.g., Kendon, 1967; Emery, 2000; Rogers et al., 2018) and also by examining the data we have collected. We considered the following factors while creating the tag set including 14 labels:

- Separate labels were identified for *Speech, Asking a Question*, and *Confirmation*.
- We classified pauses by their duration as proposed by Heldner and Edlund (2010) (*Pre-Speech, Speech Pause, Micro Pause*).
- In parallel with the turn management role of speech, we defined separate label for *Signaling End of Speech*.
- We named the conversation segment as *Thinking* when it included filler sounds, such as uh, er, um, eee, and drawls.
- As the interviewer reads the questions from the screen, the interviewer's gaze would evidently be directed toward the screen, so we tagged this case separately (*Read Question*).
- A separate label for repeating the question was identified (*Repetition of the Question*).
- We assumed that gaze direction would be affected by laughter (*Laugh, Speech While Laughing*).
- We handled *Greeting* apart from *Speech*, because we assumed that the sender would aim to signal intimacy while greeting and this might have an effect on gaze direction.
- The interviewers evaluated the interviewee's answer before proceeding to the next question. This evaluation process was performed by looking at the screen. If it did not meet one of the above conditions, the interval from the end of the interviewee's answer to the beginning of the new question was labeled as *Questionnaire Filling*.

The second method is dialogue act analysis using the ISO 24617-2 standard. The closer the microphone was to the participant, the cleaner and the better the gathered audio recording was. Therefore, in order to not miss any data, we transcribed the conversations by listening to the audio streams of both the interviewer and the interviewee in a pair separately. We first opened a Google Document and enabled speech to text feature, then started to articulate audio while listening to the interviewee's audio stream. After that, we listened to the same recording once more to add non-verbal vocalizations to the transcribed texts, such as Unfinished Word, Filler Sound, Laugh, Drawl, Warm-up, and so on. Adding non-verbal vocalizations is recommended by the ISO 24617-2 standard depending on their effect on the choice of communicative function, or qualifiers (ISO 24617-2, 2012; Bunt et al., 2017a). Then, while we were listening to the interviewer's audio stream for the same pair, we completed missing words in the transcription text file of a session. Thus, we reviewed the transcription of a session twice in this phase. Lastly, we divided the transcription text file into two separate files based on the source. As a result, at the end of the Transcription phase, two files per session were created in total, one for the interviewer's transcription and other for the interviewee's.

Secondly, by using the Praat^6^ program, three students marked the time interval of a total of 16,716 words in 15 out of 25 sessions. When selecting these 15 sessions, we have given priority to long sessions in which dialogue act and RR tagging might be more frequent. Praat is a free application for speech analysis in phonetics. We employed only the “Transcribing speech with Praat function.” As we have already transcribed audio stream, the word or non-word vocalization was copied from the transcription file and pasted into the related area in an interface. Then, the time interval of a word was specified by marking the beginning and the end. Even though we reviewed the transcript text twice in the previous phase, there would still be some missed words or non-word vocalizations. In such cases, the transcription file was updated with the missing word and/or non-word vocalization. In addition to that, after each word was processed, a controller checked if it was necessary to update the time intervals of words and transcribed texts. Thus, the transcribed text file was reviewed four times in total since its creation and word intervals were checked twice. As a result, at the end of this phase, we are left with a single transcription file and two files storing time intervals of words, one for the interviewer's transcription and the other for the interviewee's.

We segmented speech utterances into dialogue act units. As proposed by Prasad and Bunt (2015), dialogue act units were determined based on the meaning rather than the syntactic features. Dialogue act represents the communicative function that serves in a dialogue to change the state of mind of an addressee by means of its semantic content.

Since we were investigating the relation between dialogue act units and gaze direction, which was able to change quite fast, we specified dialogue act units in smaller intervals that differed from the previous and the subsequent dialogue act units in terms of communicative function, qualifiers, and RRs. Even though ISO 24617-2 supports RR annotation, it does not specify any particular set for RR. Thus, we employed another standard recommended by ISO 24617-2 for the annotation of discourse relation. ISO 24617-8, also known as ISO DR-Core, was proposed as an international standard for the annotation of discourse relations (Prasad and Bunt, 2015; Bunt and Prasad, 2016; ISO 24617-8, 2016). To understand the discourse as a whole, the relation between the sentences or clauses in the discourse (i.e., Rhetorical Relations) should be considered.

**Figure 2:**
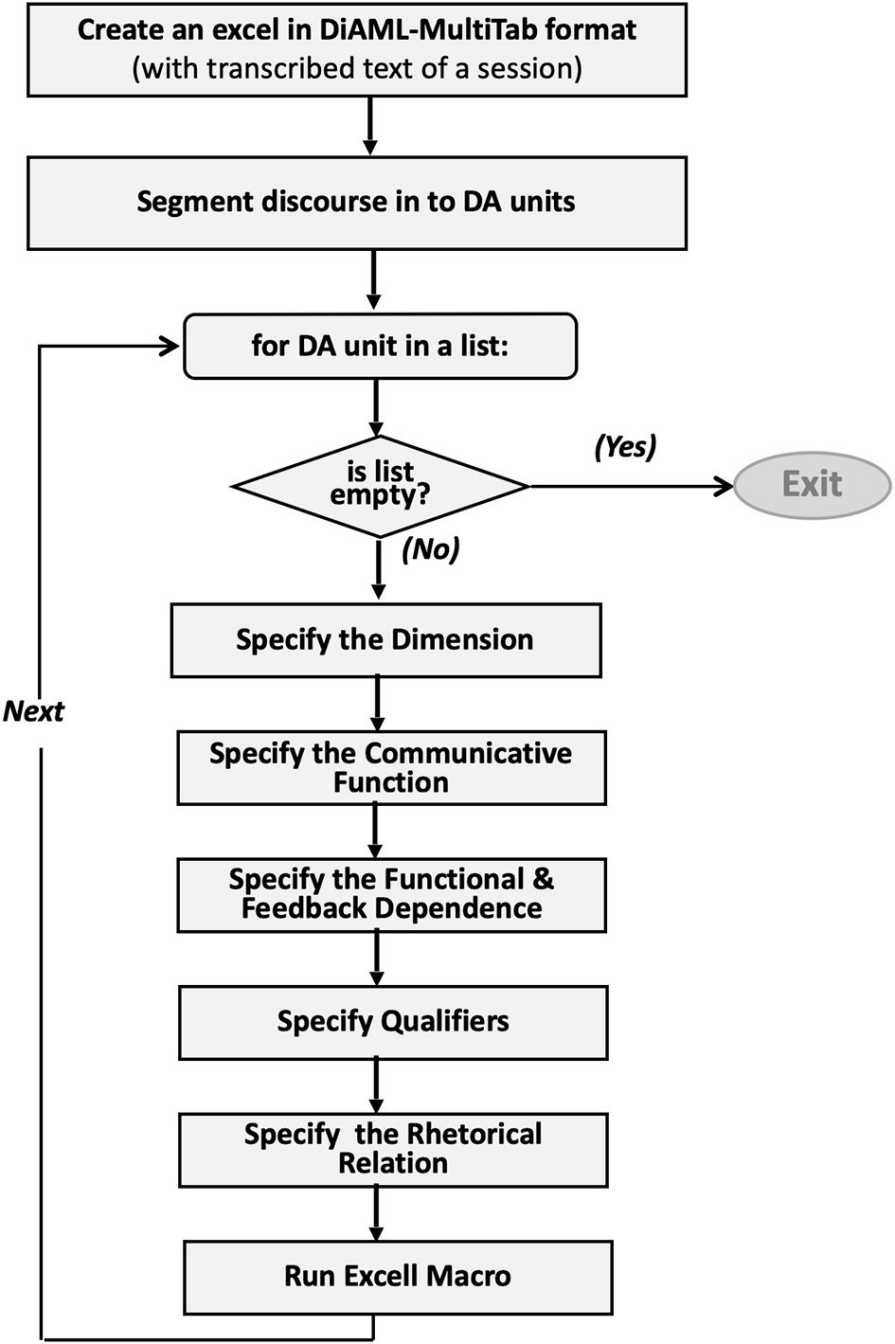
The workflow of segmentation and annotation.

Lastly, dialogue act units were annotated on the human-friendly excel file in DiAML-MultiTab format; the workflow is presented in Figure 2. According to DiAML-Multitab representation, an annotator has to assign the unique ID to each dialogue act. Moreover, if there is a functional or feedback dependence between two dialogue acts, intending to represent this relation, the ID of the preceding dialogue act should be referenced by the succeeding one. We developed an excel macro^7^ to automatize the process of assigning unique ID's and updating references. As suggested in the annotation guideline, whatever the way the speaker expressed himself, the following questions were considered during annotation: (i) why the speaker said it, (ii) what the purpose of the speaker in using this utterance is, and (iii) what the speaker's assumptions about the person he was addressing are. ISO 24617-2 indicates that labeling should be based on the speaker's intention, instead of what he or she says literally. Therefore, this standard proposes to think functionally rather than relying on linguistic cues, which are useful but focusing only on them could make us miss what the speaker really wants to say and that would cause false labeling^8^.

ISO 24617-2 proposed nine dimensions based on the type of semantic content: Task, Turn Management, Time Management, Auto Feedback, Own Communication Management, Discourse Structuring, Social Obligation Management, Allo Feedback, Partner Communication Management, and 56 communicative functions. In the present study, we encountered 43 out of 56 communicative functions, except the following ones: Correction, Accept Offer, Decline Offer, Decline Request, Decline Suggestion, Auto Negative, Allo Negative, Feedback Elicitation, Return Self Introduction, Question, Address Offer, Address Request, and Address Suggest. Moreover, ISO DR-Core recommends 18 labels for RR annotation. In the present study, all 18 labels were included.

We calculated the intra-annotator agreement via Cohen's Kappa score to measure annotation (or annotator) reliability. More than 6 months after the first annotation, the same annotator annotated ~25% of the data (corresponding to six sessions out of 25 sessions for annotations with speech tag set and four sessions out of 15 sessions for annotations with ISO 24617-2 standard). The Cohen's Kappa scores were observed to be equal to 0.85, 0.80, and 0.89 for dimensions of ISO 24617-2, communicative functions of ISO 24617-2 and speech tags, respectively (*p* < 0.0001).

#### Gaze Analysis

We performed gaze analysis by using the related interfaces of developed application (Arslan Aydin et al., 2018). Firstly, we exported raw data of eye movements as an output file storing *x* and *y* positions of the right eye at a resolution of 33.3 ms.

Then, in order to interpolate missing gaze data, first the scaling factor was calculated via Equation 1 (where *t* represents timestamp), and then the location of the first sample after gap was multiplied by the scaling factor, and lastly the result was added to the location of the last sample before the gap. The max gap length that would be filled with interpolation was chosen to be shorter than a normal blink, which was 75 ms as proposed by previous studies (e.g., Ingre et al., 2006; Komogortsev et al., 2010; Benedetto et al., 2011).

(1) $$\begin{array}{l} s_{scaling\;factor}=\frac{t_{sample\;to\;be\;replaced\;}-\;t_{first\;sample\;after\;gap}}{t_{last\;sample\;before\;gap}\;-\;t_{first\;sample\;after\;gap}}, \end{array}$$

[(1), taken from Olsen, 2012]

**Figure 3:**
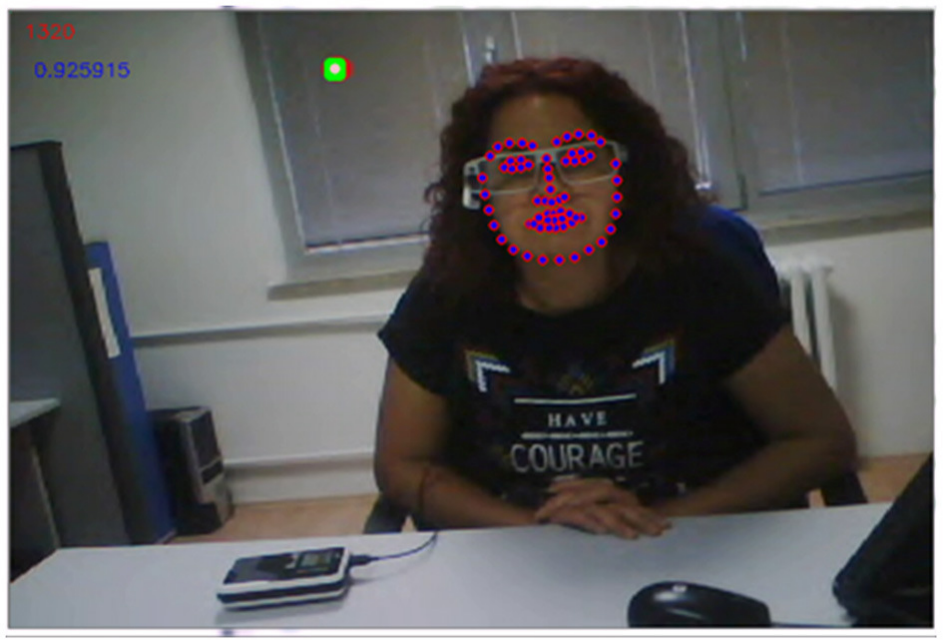
The set of facial landmarks are presented around the face of the interviewer with pink circles. The green dot represents the gaze location of the conversation partner. Color should be used in print.

Secondly, we extracted face boundaries with the default detector proposed by the developed application. Video recordings of 28 pairs consisting of a total of 828,618 frame images were processed for gaze analysis. The face boundaries over 68 2D facial landmarks were automatically detected and stored under text files as an outcome of face-tracking process. Thirdly, we extracted Area of Interest (AOI) labels corresponding to the frame image, along with the input parameters: (i) 2D landmarks of faces; and (ii) linearly interpolated raw gaze data. AOIs provided information of whether, at a particular time, a participant was looking at the interlocutor's face, i.e., face gaze, or looking away from it, i.e., aversion. Also, the relative positions of gaze data with respect to the face on each particular frame image were stored. If the gaze position was outside the face boundary, one of eight character values, a, b, c, d, f, g, h, and i, was assigned in order to denote gaze aversion; otherwise, an e character was assigned as an AOI label to denote face gaze (see Figure 3).

Fourthly, we monitored the efficiency of face detection by looking at the number and percentage of extracted AOI labels in frame images. The detection of AOI labels failed due to undetected faces and/or the missing gaze data. Fifthly, we trained a custom face detector via training interface of the developed application for the video streams in case more than 30% of frame images could not be assigned to an AOI label. Then, we extracted face boundaries with the custom detector and, after that, monitored the performance. The detection percentage of AOIs that were extracted by employing either default or custom face detector were compared, and we continued the analysis with the AOIs that got the higher detection ratio. We carried on analysis for 11 records of interviewees and a single record of interviewers with AOI labels extracted by employing trained detectors. For all remaining recordings, the ones extracted by employing default detectors were adopted. Sixthly, we assigned AOI labels to the frame images manually for the following cases:

- The face of the interlocutor was on frame image, yet it could not be detected automatically.
- The face of the interlocutor was on frame image, but it was not detected correctly.
- The face of the interlocutor did not exist for that particular frame image. This happens especially when an interviewer was looking at the monitor while evaluating the response or reading the question. In such cases with respect to the location of monitor, we easily inferred AOI label.

After reviewing and updating the extracted AOI labels manually, we re-monitored the performance and eliminated three pairs in which the amount of assigned AOI labels correspond to <70% of interviewers' and/or interviewees' recordings in a pair. Hence, we continued analysis with the remaining 25 pairs.

**Figure 4:**
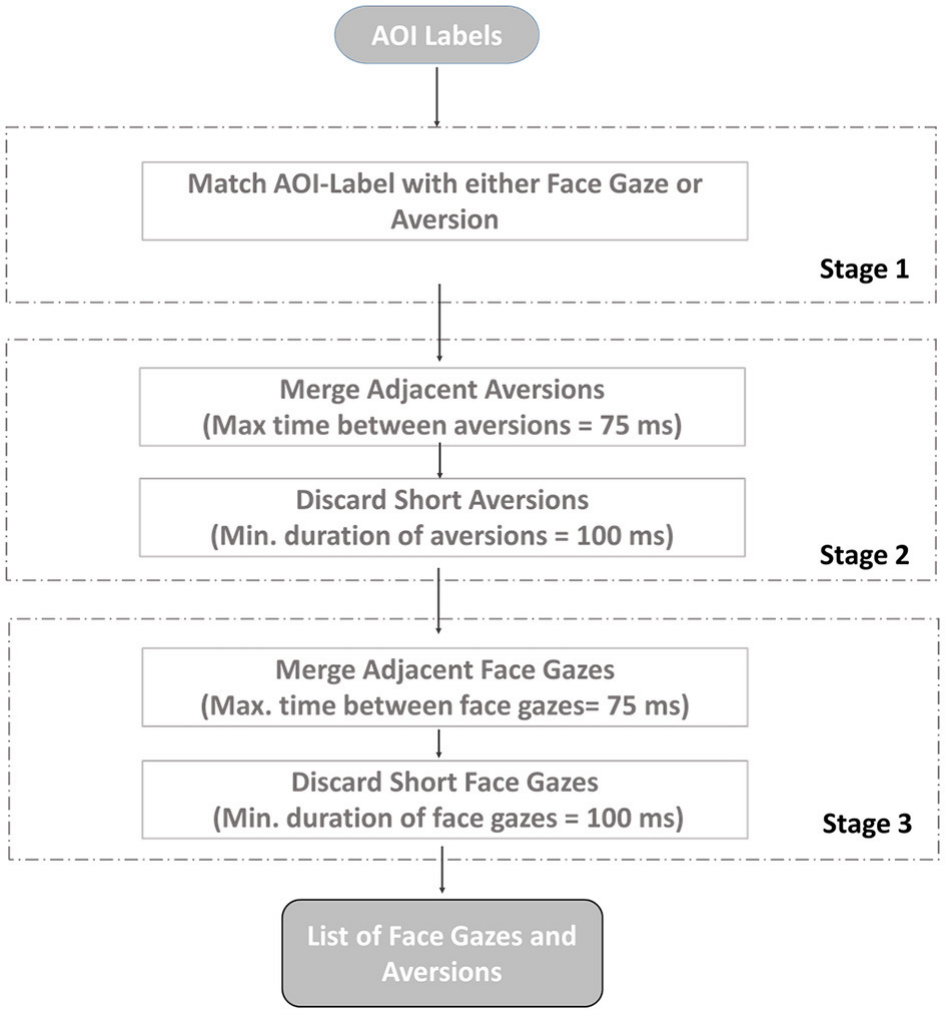
Process flow for fixation detection of gaze directions.

Lastly, in order to get rid of noise, saccadic movements, or blinks in the data, fixations were extracted in order to group the raw gaze data. In line with the literature (e.g., Manor and Gordon, 2003; Camilli et al., 2008; Komogortsev et al., 2010; Benedetto et al., 2011), we followed the consequent steps, as illustrated in Figure 4.

#### Multimodal Data

For each speech annotation method, the data obtained in speech and gaze analyses were merged into a single summary file. As a result, we obtained a series of gaze direction and related features taken at successive intervals of 33.3 ms.

##### Gaze and Speech Tag Set

The columns of the summary file were the speech tag, sender, gaze direction of sender, and of an interlocutor on the particular frame image.

##### Gaze and Dialogue Act

We first found the time interval of a particular dialogue unit by concatenating the time intervals of each word that produced a dialogue unit together. In the summary file, each line represented the gaze direction of a sender and of an interlocutor on the particular frame with the corresponding communicative function(s), dimension(s), sender information, and, if exist, RR(s), functional dependence(s), feedback dependence(s), certainty, and sentiment qualifier.

## Analysis Results

All statistical analyses were carried out in R programming language (R Core Team, 2016) and publicly available^9^. We first screened data and removed outliers. After that, we checked the assumptions of analysis and consequently decided whether we should transform data and run the parametric test or the non-parametric one. We handled individual differences by employing mixed models.

### Frequency

**Figure 5:**
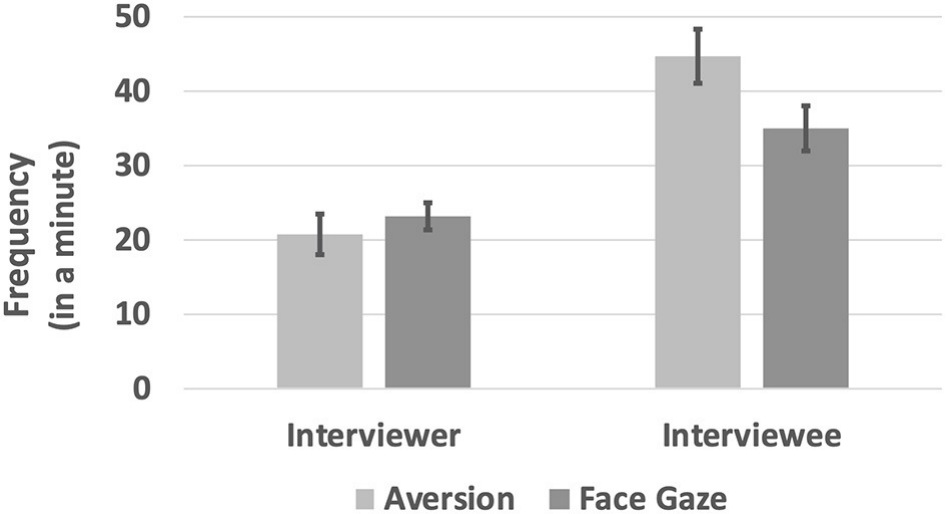
The frequency of gaze directions per roles in a minute.

We calculated the normalized frequency by dividing the count of extracted AOIs of a particular session by the duration of that session. The paired sample *t*-test was performed to compare the frequencies of face gaze and aversion per role. The analysis revealed that there was no significant difference between the frequencies of gaze aversion (*M* = 20.8, *SE* = 2.62) and face contact (*M* = 23.2, *SE* = 1.86) for interviewers, *t*_(22)_ = −1.82, *p* = 0.08. On the other hand, interviewees' gaze aversion frequency (*M* = 44.7, *SE* = 3.6) was significantly higher than their face contact frequency (*M* = 35, *SE* = 3.13), *t*_(24)_ = 2.49, *p* = 0.02. Moreover, interviewees performed aversion (*M* = 44.7, *SE* = 3.60) and face gaze (*M* = 35, *SE* = 3.13) more frequently compared to the interviewers (aversion: *M* = 20.8, *SE* = 2.62; face gaze: *M* = 23.2, *SE* = 1.86) and the differences were significant for both aversion, *t*_(23)_ = −5.03, *p* < 0.000, and face gaze, *t*_(22)_ = −3.28, *p* = 0.003 (see Figure 5). It is possible for an interviewer to perform higher frequency in both gaze directions. Because there was also significant difference in the duration of gaze directions between roles, see section Duration.

We conducted analysis with the fixations instead of raw gaze data. Raw gaze data include noise and saccadic movements, which are rapid and designed to direct the fovea to the vision of interest. Saccadic behavior might be important for particular research questions like searching for visual targets, but in the present study, since we focused on maintaining gaze on the interlocutor's face or out of the face, we should eliminate jumping behaviors as well as noise from the data.

In this study, if we had worked with raw gaze data instead of fixations, we could not observe a significant effect of the role on the frequency of gaze directions. The average frequency of face gaze comprised 53% of the sessions for interviewers, whereas it was 58% for interviewees. We also examined the frequency and duration of when two participants look at each other's face at the same time, i.e., mutual face gaze. The mutual face gaze averagely comprised 27.7% (*SE* = 4.51) of the entire session, and its average duration was 517.7 ms (*SE* = 0.23).

### Duration

We first screened data and removed outliers, and then tested the assumptions of the linear mixed model. Since the data were non-normal and violated the homogeneity assumption, we performed penalized quasi-likelihood (PQL) instead of linearity test. PQL is a flexible model that can deal with unbalanced design, non-linear data, and random effects.

We compared the potential models by ANOVA test to find out which one fits best. The statistical model for the duration of gaze aversion is given in Equation 2 below. Fixed effects were *Gender, Partner Gender, Role*, and their two-way and three-way interactions. In addition to that, the mixed effect term was added for varying intercepts by interviewers, and by interviewees that are nested within interviewers' groups. Lastly, we considered varying the slope of the interaction between *Gender* and *Partner Gender* differing across interviewers' groups.

(2) $$\begin{array}{r} \text{Fixedeffects}=\text{Role }\times \text{ Gender }\times \text{PartnerGender},\\ \text{Randomeffects}=1+\text{Gender }\times \text{PartnerGender}\\ |\text{InterviewerID}/\text{IntervieweeID}. \end{array}$$

The statistical model for the duration of face gaze is given in Equation 3. We compared the potential models by ANOVA test to find out which one fits best. Fixed effects were *Gender, Partner Gender, Role*, and their interactions. In addition to that, the mixed effect term was added for varying intercepts by interviewers, and by interviewees that are nested within interviewers' groups.

(3) $$\begin{array}{r} \text{Fixed effects }=\text{ Role }\times \text{ Gender }\times \text{ PartnerGender},\\ \text{Random effects }=\;1|\text{ InterviewerID }/\text{IntervieweeID}. \end{array}$$

**Figure 6:**
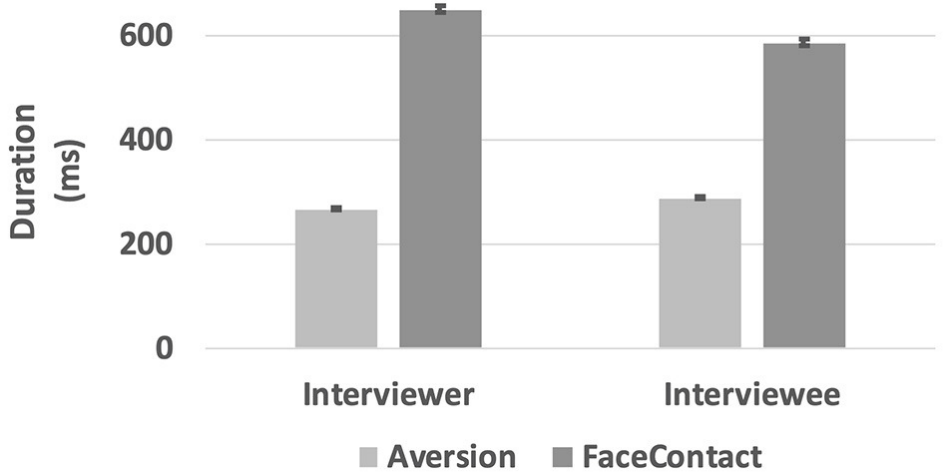
The average duration of gaze directions in ms.

The interviewer's face gaze duration (*M* = 648.9 ms, *SE* = 7.06) was significantly higher than the interviewee's face gaze duration (*M* = 585.8 ms, *SE* = 6.06), *t*_(10, 434)_ = – 1.977, *p* = 0.048. There was a significant effect of the role, i.e., being an interviewer or an interviewee, on the duration of gaze aversion (see Figure 6). The *post-hoc* tests revealed that a significant difference between the aversion durations of interviewers (*M* = 258.2 ms, *SE* =5.25) and interviewees (*M* = 313.2 ms, *SE* = 3.43) was observed when the partner gender was female, *t*_(9, 760)_ = 5.75, *p* < 0.0001.

### Multimodal Analysis of Gaze and Speech

In multimodal analysis, we examined the relation of gaze direction with either speech tags or communicative functions. The statistical analyses were conducted on the top five labels for both annotation schemes. In this section, we will describe the analysis steps via speech tag set. Similar calculations were also performed for dialogue act analysis.

Primarily, we extracted the ratio of gaze behavior observed during an instance of speech tag set. Each instance of speech tag set might be assigned several times during a session. In Equation 4, let *B* be a set including percentages of aversion (*A*) and face gaze (*FG*) during occurrences of speech tags, for session *x* and participant *p*, where *i* is the element of *F*, which is a set of frame IDs labeled with particular speech tag. *D* function gets the frame IDs and type of gaze direction, namely, *A* or *FG*, as input parameters and returns the durations of that specified gaze direction among those frames.

(4) $$\begin{array}{r} B_{x,p}\left(S,A\right)=\left\{i\;\epsilon \;F_{s}:\frac{D\left(i,A\right)}{D\left(i,A\right)+D\left(i,FG\right)}\right\}, \end{array}$$

The process details are given in Table 3. A sample implementation of Equation 4 for Table 3 would be as follows:

**Table 3:** Illustration of calculating the ratio of gaze direction (GD) to the particular speech-tags, S_1,#index_ and S_2,#index_.

**Frame no**.	**Speech-Tag(S) (_**id,#index**_)**	**GD**	**Ratio of GD duration**
1	S_1,1_	A	|A| / |S_1,1_|= 6/9
2		A	
3		A	
4		A	
5		A	
6		A	
7		FG	|FG| / |S_1,1_| = 3/9
8		FG	
9		FG	
26	S_2,1_	FG	|FG| / |S_2,1_|= 10/20
27		FG	
28–35		FG	
36–44		A	|A| / |S_2,1_|= 10/20
45		A	
46	S_1,2_	A	|A| / |S_1,2_| = 25/50
47		A	
48–70		A	
71		FG	|FG| / |S_1,2_| = 11/50
72–81		FG	
82		A	|A| / |S_1,2_|= 14/50
83–95		A	

*Only the interviewer's gaze behavior is considered. A similar calculation is also performed for interviewees. We intentionally skipped the frames between 10 and 25 to simulate realistic data. During the analysis, we excluded the frames in which there was no extracted gaze direction for the interviewer or interviewee*.

Frame Set:

*F*_*s*1_ = {[1–9], [46–95]}

*Gaze Directions*:

*D*([1−9], *A*) = {6}; *D*([46−95], *A*) = {25, 14}

*D*([1−9], *FG*) = {3}; *D*([46−95], *FG*) = {11}

*Set of Aversion Percentages, during S*_1_:

*B*_1,*interviewer*_(*S*_1_, *A*) = {i ϵ {[1–9], [46–95]}: $\frac{D\left(i,A\right)}{\left(D\left(i,A\right)+D\left(i,FG\right)\right)}$ }

*B*_1,*interviewer*_ (*S*_1_, *A*) = {6/9, 25/50, 14/50}

*Set of Face Gaze Percentages, during S*_1_*:*

*B*_1, *interviewer*_ (*S*_1_, *FG*) = {i ϵ {[1–9], [46–95]}: $\frac{D\left(i,FG\right)}{\left(D\left(i,A\right)+D\left(i,FG\right)\right)}$ }

*B*_1, *interviewer*_ (*S*_1_, *FG*) = {3/9, 11/50}

As well as the duration, we also calculated the frequency of gaze directions during a particular speech tag. This time, we just consider the fixation counts of related gaze direction. For instance, in Table 3, the frequency of face gaze was one for S_1, 2_, whereas the frequency of aversion was two. Thus, the percentages were 1/3 and 2/3, respectively.

#### Speech Tag Set Annotation

The data were non-normal and violated the homogeneity assumption; thus, we performed PQL. The statistical model is described by Equation 5. Fixed effects were *Role, Speech tag*, their mutual interaction, *Interviewer's Gender, Interviewee's Gender*, and their mutual interaction. Besides, the mixed effect term was added for varying intercepts by interviewers and by interviewees that are nested within interviewers' groups. Lastly, we added the *Speech tag ID*, which was a unique identifier for each occurrence of speech tag, as a mixed effect term.

(5) $$\begin{array}{r} \text{Fixed effects }=\text{ Role }\times \text{SpeechTag }+\text{ Interviewe}{\text{r}}^{\text{′}}\text{s Gender}\\ \times \text{ Interviewe}{\text{e}}^{\text{′}}\text{s Gender},\\ \text{Random effects }=\;1|\text{ InterviewerID}/\text{IntervieweeID }+1|\\ \text{Speech tag ID}. \end{array}$$

**Figure 7:**
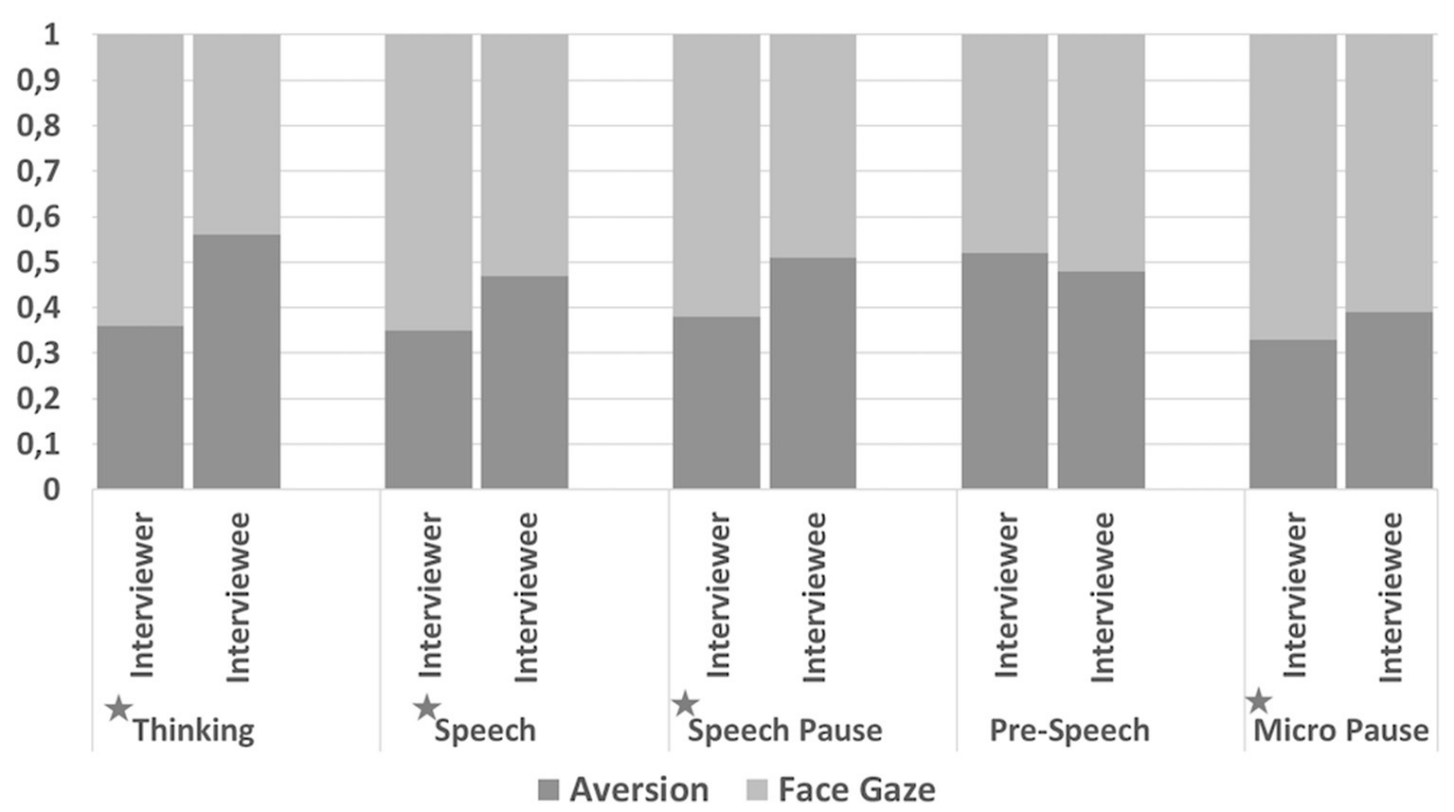
Frequency of gaze direction ratios for the top five speech tags observed in the collected data. Since the gaze direction can be either face gaze or aversion, a total ratio for all bars are 1. Significant differences are presented with * character.

There was a significant difference in the frequency of gaze direction ratios between the interviewers and interviewees when the speech tag was *Thinking* [*t*_(6, 840)_ = 13, *p* < 0.0001], *Speech* [*t*_(6, 840)_ = 12.9, *p* < 0.0001], *Speech Pause* [*t*_(6, 840)_ = 10.8, *p* < 0.0001], or *Micro Pause* [*t*_(6, 840)_ = 7.23, *p* < 0.0001] (see Figure 7).

We also examined the difference in duration of gaze direction between the interviewers and interviewees. Similarly, results revealed that when the speech tag was *Thinking* [*t*_(6, 840)_ = 13.3, *p* < 0.0001], *Speech* [*t*_(6, 840)_ = 12.9, *p* < 0.0001], *Speech Pause* [*t*_(6, 840)_ = 10.7, *p* < 0.0001], or *Micro Pause* [*t*_(6, 840)_ = 7.8, *p* < 0.0001], interviewee's gaze aversion duration was significantly longer than the interviewer's.

#### Dialogue Act Annotation

The data were non-normal and violated the homogeneity assumption; thus, we performed PQL. The statistical model is described by Equation 6. Fixed effects were *Role, Communicative Function*, their mutual interaction, *Interviewer's Gender, Interviewee's Gender*, and their mutual interaction. In addition, the mixed effect term was added for varying intercepts by interviewers and by interviewees that are nested within interviewers' groups. Lastly, we also added the *Communicative Function ID*, which was a unique identifier for each occurrence of communicative functions, as a mixed effect term.

(6) $$\begin{array}{r} \text{Fixed effects }=\text{ Role }\times \text{Communicative Function}\\ +\text{ Interviewe}{\text{r}}^{\text{′}}\text{s Gender }\times \text{ Interviewe}{\text{e}}^{\text{′}}\text{s Gender}\\ \text{Random effects }=\;1|\text{ InterviewerID}/\text{IntervieweeID }+1|\\ \text{Communicative Function ID}. \end{array}$$

**Figure 8:**
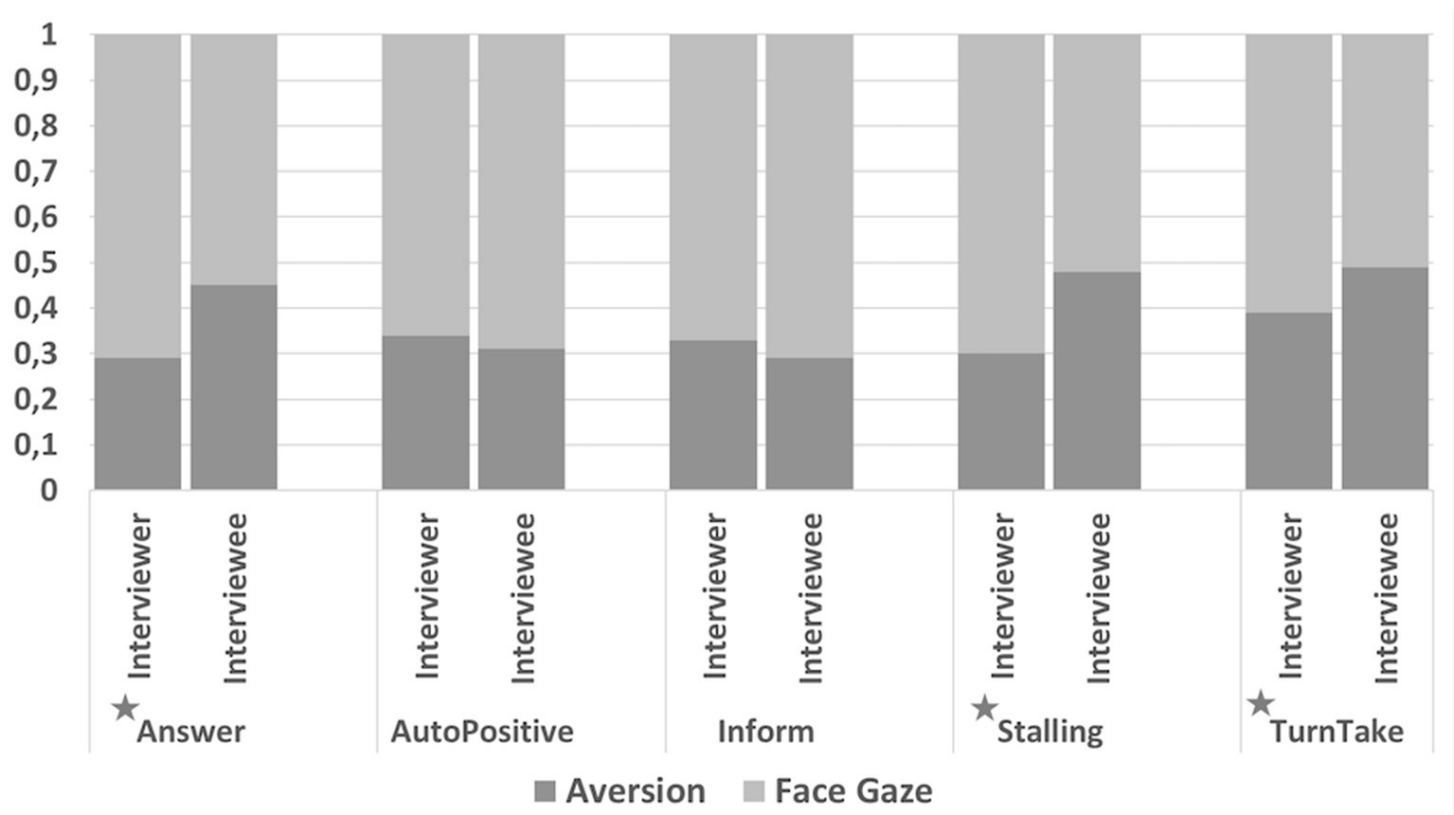
Frequency of gaze direction ratios for the top five communicative functions observed in the collected data. Since the gaze direction can be either face gaze or aversion, a total ratio for all bars are 1. Significant differences are presented with * character.

There was a significant difference in the frequency of gaze direction ratios between the interviewers and interviewees when the communicative function was *Answer* [*t*_(5, 334)_ = 13.1, *p* < 0.0001], *Stalling* [*t*_(5, 334)_ = 19.9, *p* < 0.0001], or *Turn Take* [*t*_(5, 334)_ = 5.69, *p* < 0.0001] (see Figure 8).

We also examined the difference in the duration of gaze direction between the interviewers and interviewees. Similarly, results revealed that when the communicative function was *Answer* [*t*_(5, 334)_ = 14.2, *p* < 0.0001], *Stalling* [*t*_(5, 334)_ = 19.8, *p* < 0.0001], or *Turn Take* [*t*_(5, 334)_ = 5.58, *p* < 0.0001], interviewee's gaze aversion duration was significantly longer than the interviewer's.

## A Deep Computational Model

For computational modeling, we use CNNs. CNNs are specialized versions of fully connected networks with localized receptive fields. In the present study, we adapted simplified versions of two state-of-the-art CNN architectures, namely, ResNet (He et al., 2016) and VGGNet (Simonyan and Zisserman, 2015).

**Figure 9:**
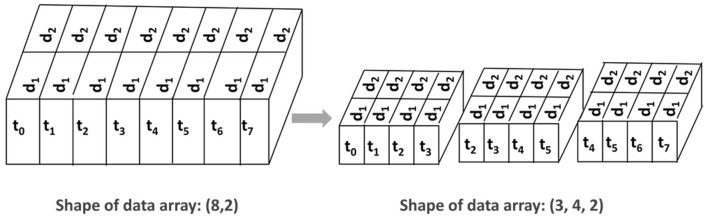
The time-series data and how they are prepared before processing with the deep networks. On the left, the input has a size of 8 × 2 where the number of time steps is eight and the number of channels is two. On the right-hand side, the shape has changed to 3 × 4 × 2 where the window size is four and the stride is two.

We collected gaze data in the form of a time series and trained two 1D CNN networks. In 1D CNNs, data points in time series are generally introduced to the network as a window of instances. The window is slid in time by a number of time steps, which is called stride. For instance, for a two-channel signal consisting of eight time steps, a window size of four and stride of two would yield three input samples with a size of 4 × 2 (see Figure 9).

**Figure 10:**
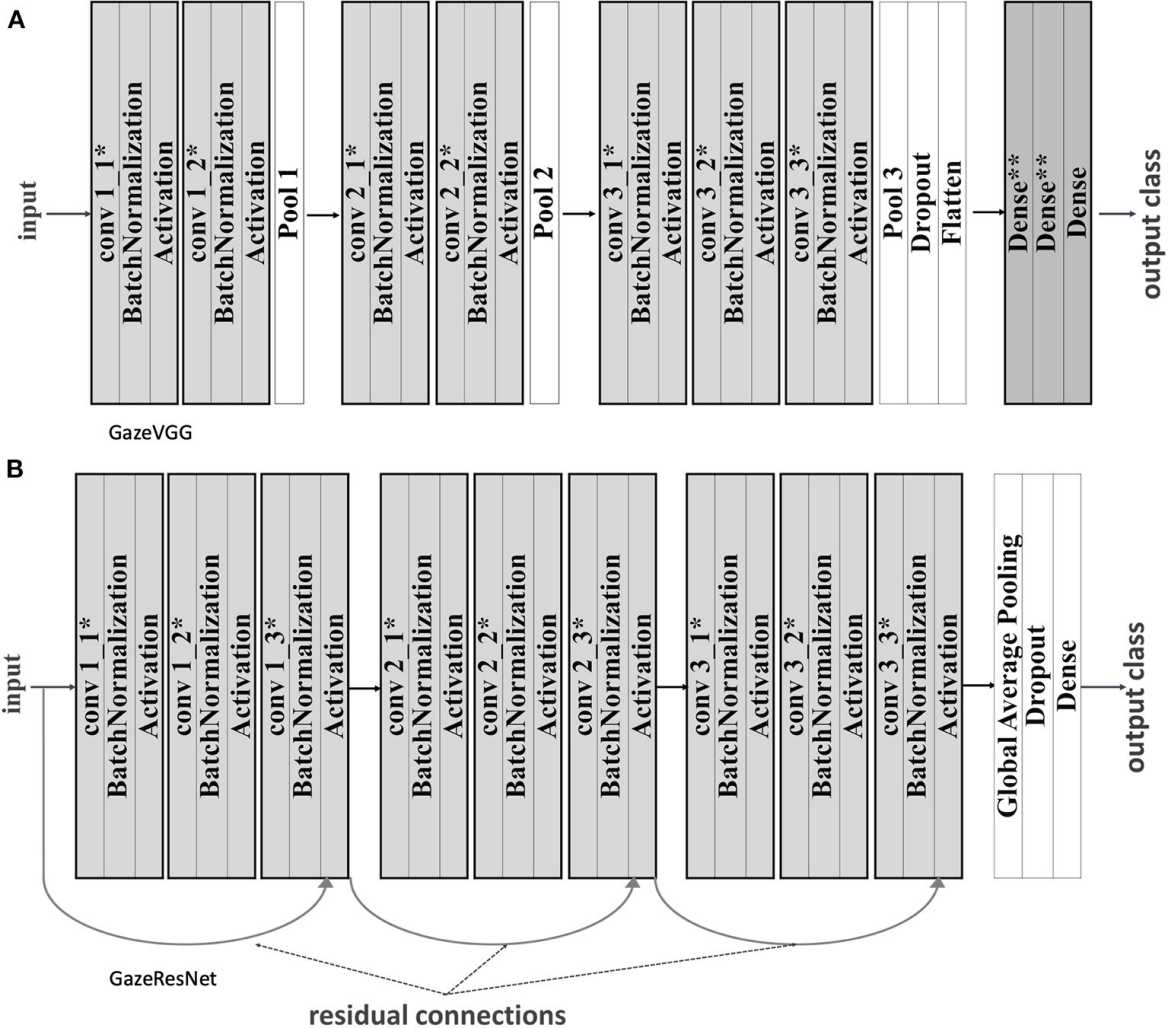
**(A)** GazeVGG architecture with batch normalization, regularization, and pooling. **(B)** GazeResNet architecture with batch normalization, regularization, and pooling. There is convolution between blocks and a residual connection between the last item of the previous block and the current one. *L2 weight and bias regularizers were applied. **L2 weight regularizer was applied.

We adapt two CNN architectures (VGGNet and ResNet) and called them gazeVGG and gazeResNet (see Figure 10). Batch normalization, pooling, weight regularization, and dropout were applied to both networks for handling overfitting.

### Data Presentation Details

In the present study, we obtained a series of gaze direction and related features at successive intervals of 33.3 ms. According to the data obtained from the human–human experiment (see section Experimental Investigation), the average gaze aversion duration was ~300 ms. Therefore, we used nine as the window size as single frame took 33.3 ms, and since the minimum fixation duration was 100 ms, we set stride to three.

In our experimental design, while the interviewees participated in a single session, interviewers took part in multiple interviews. We designed our computational models for predicting gaze direction of interviewers. At first, we applied One-Hot-Encoding to convert categorical data into numbers. For the input data including speech annotation with the speech tag set, we used a total of 20 features including *Sender, Speech Instance, Gender, Is the Same Person*, and *Interviewee's Gaze direction*. On the other hand, a total of 137 channels involving *Sender, Gender, Is the Same Person, Interviewee's Gaze direction, Communicative Function, Dimension, Certainty, Sentiment, Functional Dependence, Feedback Dependence, Rhetorical Relation*, and *Argument Number of Rhetorical Relations* were utilized for the dialogue act models. Therefore, for a window of size nine, a single input to a CNN had 180 dimensions for data annotated with the speech tag set and 1,233 dimensions for data annotated with ISO 24617-2 standard.

### Training and Implementation Details

For both CNNs, binary-cross entropy was used as the objective function, which was minimized using Adam optimizer. Moreover, we used dropout with a value of 0.2 and L2 regularization with a value of 0.001. We trained gazeResNet models for 100 epochs with a batch size of 64. Similarly, we trained gazeVGG models for 100 epochs with a batch size of 64 and pool sizes of 2. We have empirically changed and evaluated the different settings for L1, L2, epoch count, window size, stride, etc., and we have provided the best settings. In the hyper-parameter tuning phase, we used backtesting that is specific to the time series as a cross-validation method. We trained gazeVGG and gazeResNet models with 16 or 32 filters in the first block and, taking input data, annotated either with an ISO 24617-2 standard (i.e., dialogue acts) or speech tag set. For the data annotated with dialogue acts with 32 filters in ResNet and 16 filters in VGG, and for the data annotated with a speech tag set, 32 filters in both VGG and ResNet achieved better accuracies.

In the *n*-fold back-testing, the ratio of data provided for the training and validation is different at each split. It is five for the fifth split and one for the first split. When the training data are not big enough, the network might not quite learn about the underlying trend of the data. For instance, in the present study, the second and the third interviewer had a greater tendency to aversion whereas the sixth one had a tendency in the opposite direction. Hence, especially for the second and the fourth splits, the distribution of data for training and testing was different, which resulted in validation fluctuations. Particular orders of interviewers in the input data result in specific orders of interviewers in splits used for training and validation. This might cause testing the network with a different distribution than the one used in training. The classical cross-validation method enables one to handle such distribution issues by randomly dividing the set of input data into training and test sets. However, time-series data have temporal relations that prevent randomized division. In order to overcome this issue, we trained and evaluated the models by building 5-fold cross-validation with data sets created by shuffling the orders of interviewers in the input data while preserving temporal order within each session.

Training was performed on Google Colab, which is a free Jupyter notebook environment provided by Google. Colab offers Tesla K80 GPU. The training codes were implemented in Python 3.0 by Keras libraries with Tensorflow backend.

### Results

We analyzed the gaze prediction performances of the two CNN architectures. Table 4 lists the performances with both dialogue act and speech tag inputs. We see that models running on the data annotated with speech tags generally perform better than the ones running on the data annotated with dialogue acts.

**Table 4:** Performances of computational models with 5-fold cross-validation.

**Tagging scheme**	**CNN architecture**	**Avg. training accuracy (%)**	**Test accuracy of folds (%)**	**Avg. test accuracy (%)**
Dialogue act	*VGG*	83.2 (*SD*: 1.20)	89.5, 76.7, 70.6, 60.3, 57.1	69.6 (*SD*: 11.3)
	*ResNet*	83.1 (*SD*: 0.88)	87.7, 77.1, 70.8, 59.8, 58	70.7 (*SD*: 12.3)
Speech tag set	*VGG*	81.1 (*SD*: 0.18)	83.2, 68.6, 81.5, 76.9, 74.6	76.9 (*SD*: 5.82)
	*ResNet*	81.1 (*SD*: 0.14)	**84.6, 69.6, 81.4, 82, 76.2**	**78.8** (*SD*: 5.94)

*The highest test accuracy was obtained with the GazeResNet model when applied on data annotated with the speech tags. Those accuracy values are presented in bold*.

In order to examine the quantitative differences between classification accuracy of the models, we also analyzed confusion matrices in Table 5, which contain the percentages of false and correct estimations. We notice that models with both speech tag and dialogue act could predict the direction of face gaze with similar and relatively high accuracies (i.e., speech tag set model achieved 85.1% accuracy and dialogue act model achieved 94.8% accuracy), whereas there was a difference in the prediction accuracies of aversion between the models. Speech tag set model could predict aversions better than Dialogue act model.

**Table 5:** Confusion matrix of the GazeResNet models with the highest performances for each tagging scheme.

	**Predicted class**		
	**Speech tag set/Dialogue act (%)**		
		**Face gaze**	**Aversion**
Actual class	Face gaze	85.1/94.8	14.9/5.2
	Aversion	23.7/46.0	76.3/54.0

*It represents the percentages of true and false predictions made on actual classes, i.e., aversion and face gaze. The percentage of true aversion predictions is 76.3% for the Speech tag set model, while it is 54% for the dialogue act model*.

The performances of GazeResNet models were also assessed via calculating the recall, precision, and *F* scores. In predicting aversions, a precision of 0.69, a recall of 0.63, and an *F* score of 0.65 were obtained for the data annotated with speech tag scheme, while dialogue act scheme yielded a precision value of 0.65, a recall of 0.22, and an *F* score of 0.33.

## Discussion

Face-to-face communication is inherently multimodal. Gaze provides an effective way to receive and send information in a face-to-face interaction as a non-verbal communication channel accompanying speech. When studying gaze and speech, it is necessary to decide from which level both models will be addressed. Low-level eye movements, anatomic features of the eye, and kinematics of eye movements have been extensively studied by physiologists. However, although there exist studies in the related fields, eye movements have some other high-level characteristics that are still waiting to be resolved, like when they occur, how long they last, and what their roles are in communication (Ruhland et al., 2015). As in the gaze studies, researchers have dealt with the speech at different levels for modeling non-verbal communication components driven by speech (Cassell et al., 1999; Zoric et al., 2011; Marsella et al., 2013).

### Experimental Analysis

In the present study, we investigated the roles of the high-level characteristic of eye movements driven by high-level features of speech in a face-to-face interaction. The two major research questions of the study were: “What are the underlying features of gaze direction among humans” and “What is the relation between gaze and speech to achieve conversational goals in a specified face-to-face interaction?” To examine these questions, we conducted a mock job interview task. Twenty-eight pairs consisted of seven professional interviewers and 28 interviewees took part in the study. They wore Tobii glasses throughout the study.

We automated the analysis mostly by utilizing the state of the art methods. That way, we aimed to overcome some methodological problems and reduce the amount of human-related errors and the time necessary for annotation. We used an open source project (Arslan Aydin et al., 2018) that provided interfaces for the analysis of gaze involving face detection and identification of gaze direction. Moreover, it enabled speech analysis including segmentation, annotation, and synchronization of pair's recordings.

Gaze direction was identified as either face gaze or gaze aversion based on the decision whether the participant was looking at the other person's face or not. The gaze analysis was carried out in three steps: (i) determining the boundaries of the face, i.e., face detection; (ii) deciding whether the partner's gaze was within those boundaries, i.e., identification of gaze direction; and (iii) fixation detection.

We monitored the ratio of unidentified gaze direction on frame images of recordings. We observed that the AOI identification rate on the frame images of the 11 interviewees' recordings and two interviewers' recordings was <70%. By visualizing the recordings frame by frame, we realized that even there exist gaze raw data of interviewees, the interlocutors' (i.e., interviewers') face might not be detected while they were reading a question or evaluating the responses of an interviewee by turning their head and accordingly face to the screen. For such cases, we trained a custom face detector instead of using Haar-Cascade classifiers, which were provided by the OpenFace software, as the default detector. Moreover, in order to minimize data loss, we manually determined the gaze direction on frame images if they could not be detected automatically, but it was possible to identify their AOI labels, like in the cases when the face of the interlocutor was on frame image but could not be tracked automatically.

We observed that interviewees performed face gaze and aversion significantly more frequently when compared to interviewers. Moreover, the gaze aversion durations of interviewers were significantly longer than those of interviewees. On the other hand, face gaze durations of interviewees were significantly longer than that of interviewers. When we examined gaze direction per role, we found that there was no difference between the frequencies of gaze aversion and face gaze for interviewers, while a significant difference was observed for interviewees. Interviewees avert their gaze more frequently compared to performing face gaze. These findings are in line with the conclusions summarized by Kendon (1967) in his detailed study investigating the function of gaze in a face-to-face conversation. Kendon (1967) stated that individuals tend to look at others more frequently when listening compared to speaking and the glances of speakers would be shorter than the listeners. He had grouped the roles in the conversation as speakers and listeners. In the present study, due to the role of interviewees, they spoke more frequently than the interviewers. Comparing interviewers and interviewees, the gaze direction of the latter was more similar to that of the speakers mentioned in Kendon (1967).

Broz et al. (2012) studied mutual gaze in a face-to-face conversation with participants wearing eye-tracking devices. They observed a mutual face gaze occurring for about 46% of a conversation. Rogers et al. (2018) also conducted a dual eye-tracking study and reported that the mutual face gaze comprised 60% of the conversation with 2.2 s duration on average. On the other hand, when cumulative data of all sessions are taken into account, we found a lower ratio in the present study, which was 27.7% (*SE* = 4.51), and the average duration was 517.7 ms (*SE* = 0.23), possibly due to differences in data collection settings and analysis methods as reviewed below.

There are two crucial steps in determining mutual face gaze: (i) deciding whether the gaze of an individual was inside the face boundaries of an interlocutor, and (ii) synchronization of recordings exported from eye-trackers. Broz et al. (2012) and Rogers et al. (2018) manually annotated gaze direction in each frame. However, in the present study, interlocutor's face boundaries were detected based on 68 facial landmark points and gaze direction was generally decided automatically. Manual coding of gaze direction might be open to human-related errors. Compared to the previous studies, we employed state-of-the-art technologies for face boundary detection. Moreover, because of the hardware or operational constraints, eye-tracking devices might estimate gaze positions with deviations. Eye tracker manufacturers provide the estimated error that is specific to device in degrees for the visual angle. In the present study, we utilized the developed application (Arslan Aydin et al., 2018), which automatically considers such error margins to estimate gaze direction, to visualize gaze and face boundaries overlaid on a frame image. It is not possible to take exact error margin into account just by visualizing data without benefiting from proper scripts. For instance, Rogers et al. (2018) used 15 pixels for the size of the circle that represents the gaze position. They decided on a size of 15 pixels to achieve a balance between comfort in the manual coding process while providing distinguishable regions. In addition, using fixations instead of raw gaze data and the methods adapted for fixation extraction and synchronization of pair recordings might also affect the findings. Also, differences in eye-tracking equipment, cultures, spoken language, and experimental procedures might have an impact on the variety of the reported ratio of mutual face gaze and its duration. For instance, we performed a mock job interview task; on the other hand, the ratio of gaze directions of participants might be different in conversations without a predetermined topic.

We handled speech analysis by employing two annotation methods. In the first one, discourse and rhetorical relations were annotated with standards of ISO 24617-2 and ISO 24617-8, respectively. As a second method, we used an alternative set of speech tags that we produce based on studies in the role of eye movements in social communication and also based on our observations on the data that we collected. Our aim of annotating speech with the produced speech tag set is not to propose an alternative scheme for speech annotation but instead to investigate the characteristics of speech that produce better performance in modeling social gaze. Then we conducted analysis, to see the relation between gaze and speech. There was a significant difference in the frequency of gaze directions between the interviewers and interviewees when the speech tag was *Thinking, Speech, Speech Pause*, and *Micro Pause*. Interviewees' gaze aversion frequency was higher for all those cases. We performed similar analysis for dialogue acts. This time, we found that, there was a significant difference between the interviewer and interviewee when the communicative functions were *Answer, Stalling*, and *Turn Take*. Similarly, for all these three communicative functions, gaze aversion frequency was higher for interviewees compared to interviewers.

### Computational Models

The present study investigated further research questions to improve the methodology of multimodal analysis of communication, as follows: “How can we computationally model gaze direction with the high-level features of speech” and “How appropriate is employing discourse analysis scheme, namely, ISO 24617-2 standard, in a computational model of gaze direction?” To this aim, we trained two common Convolutional Neural Network (CNN) architectures, namely, VGGNet and ResNet. According to the experimental design, each interviewee took part in a single session whereas an interviewer attended more than one session. Therefore, we collected more data for each individual interviewer compared to an interviewee. We trained computational models to predict the gaze direction of interviewers.

We trained GazeVGG and GazeResNet models with 16 or 32 filters in the first block and, taking input data, annotated with either ISO 24617-2 standard or speech tag set. We observed that GazeResNet models achieved better accuracies for both annotation methods due to VGG bottleneck, which causes loss of generalization capability after some depth whereas ResNet handles this vanishing gradient problem by using residual connections. Moreover, we found that the speech tag set gave rise to better performances compared to dialogue act annotations. Although both GazeResNet models predicted face gaze with higher accuracies, ISO 24617-2 standard was not good at predicting aversions. Compared to data annotated with dialogue acts, Speech tags are more constant over time. Therefore, attributing the difference in the accuracy of models to that would not be a correct interpretation. The probable reasons might be the differences in the number of features and the number of input data. In addition, speech tag set involves *Pre-Speech, Speech Pause*, and *Micro Pause* for annotation of pauses whereas ISO 24617-2 standard does not handle pauses.

We obtained a series of gaze direction and related features at successive intervals of 33.3 ms in the present study. According to the human–human experiment data (section Experimental Investigation) the average gaze aversion duration was ~300 ms. Therefore, we used nine as the window size since a single frame took 33.3 ms. However, different values of window-size and stride may lead to differences in the success ratio of the models. Moreover, we just used the previous features in the training. For instance, to predict the gaze direction at t_i_, the features between t_i−8_ and t_i_ were presented to the network. However, we could get information from the subsequent frames since we conducted an offline analysis. For instance, it might be necessary to evaluate the entire speech up to t_i+10_ to decide whether the speech label at t_i_ was a *Question*. This constraint should be addressed in an online system. We think that one way to address this concern is as follows: Based on available data at the time of a prediction, confidence values might be assigned to all potential labels.

As presented in Table 4, even though we applied pooling, weight, and dropout regularizations, there was still a difference of around 10% between training and test accuracy performances of the models that receive the data annotated by ISO 24617-2 standard. To get a more robust estimation about how accurately models make predictions on unseen data, we then performed 10-fold cross-validation on those data by splitting the last 10% of data for testing in each iteration. We obtained accuracy performances similar to the 5-fold validation. Early stopping and increasing the size of input data might improve the model's generalization capability.

## Conclusion

We investigated gaze accompanying speech in a face-to-face interaction. Firstly, we studied the characteristics of gaze and its relations with speech with an experimental research conducted via mobile eye-tracking devices. The results indicate that the frequency and duration of gaze differ significantly depending on the role. We showed that these differences could not be observed in the analysis performed with raw gaze data instead of detected fixations. As in some of the previous studies, performing gaze analysis with raw gaze data or with detected fixations by using black box solutions is inadequate to obtain comparable results. Moreover, in multimodal analysis, it is important to automate annotations with the state-of-the-art methods. Manual annotation is vulnerable to human-related errors, and in addition, automatic annotation with the state-of-the-art methods provide further information that may not be extracted manually, such as detecting the coordinates of facial landmarks, taking into account the error margins while annotating the gaze direction or segmentation of the speech at milliseconds precision. In the multimodal analysis, we find the significant effect of speech tag set instances and communicative functions, those related with time and turn management, in the gaze directions.

Secondly, we developed CNN models of gaze direction in a face-to-face interaction. At the computational model of gaze, we observed that annotation with a simple tag set leads to a better performance despite the higher effort spent for making the dialogue act annotation on the same data. It might be due to the differences in the number of features and input data, but also a specific difference between the two annotation methods is whether *Pauses* are addressed. The speech tag set involves *Pre-Speech* (i.e., warming up the voice), *Micro Pause* (i.e., gaps up to 200 ms, as proposed by Heldner and Edlund, 2010), and *Speech Pause* (i.e., pauses that are not included in the other two categories) for annotation of pauses. However, the dialogue act annotation does not handle pauses. This suggests that multimodality should be taken into account when proposing automatic speech annotation schemes. Even though there was no verbal communication, *Pauses* during a conversation had an impact on non-verbal signals and, thus, on the interaction. This finding may be justified by the fact that in natural settings, listeners comprehend the speakers' messages by integrating both non-verbal and verbal channels in multiple channels (Kelly et al., 2015). In addition, results showed that CNN, especially ResNet models, allows us to predict high-level features of eye movement with high-level features of speech.

As future work, other non-verbal cues accompanying speech might be experimentally investigated to examine their characteristics, roles, and relations in social communication. In addition, the effect of language, culture, and personal differences might be investigated to assess the generalizability of the result. Moreover, neural network models mimic humanly cognitive faculty at the behavioral level. Thus, such models do not represent the process that take place in the brain. There exist articles discussing the capabilities of DNNs (e.g., Cichy and Kaiser, 2019). Despite the advances and rapid adaptation of deep neural networks in various fields, their lack of interpretability remains a problem. In particular, the visualization of 1D-CNN models that take the input data as 1D vector is relatively new; however, considering its explanatory power, future studies can be done to explore the effect of input features.

## Data Availability Statement

The datasets presented in this study can be found in online repositories. The names of the repository/repositories and accession number(s) can be found at: http://dx.doi.org/10.17632/7v728yymmk.2.

## Ethics Statement

The studies involving human participants were reviewed and approved by Applied Ethics Research Center, METU. The patients/participants provided their written informed consent to participate in this study. Written informed consent was obtained from the individual(s) for the publication of any potentially identifiable images or data included in this article.

## Author Contributions

ÜA: conceptualization, methodology, software, validation, formal analysis, investigation, data curation, writing—original draft, and visualization. SK: conceptualization, methodology, writing—review and editing, and supervision. CA: conceptualization, methodology, writing—review and editing, supervision, project administration, and funding acquisition. All authors contributed to the article and approved the submitted version.

## Conflict of Interest

The authors declare that the research was conducted in the absence of any commercial or financial relationships that could be construed as a potential conflict of interest.

## Footnotes

^1^For Draft of DAMSL: Dialog Act Markup in Several Layers, see https://www.cs.rochester.edu/research/speech/damsl/RevisedManual/.

^2^You can find a collection of dialogues annotated according to international standard ISO 24617-2 under https://dialogbank.uvt.nl/.

^3^The Sphinx4 is a speech recognition system jointly designed by Carnegie Mellon University, Sun Microsystems Laboratories, Mitsubishi Electric Research Labs, and Hewlett-Packard's Cambridge Research Lab. The Official website is: http://cmusphinx.sourceforge.net/.

^4^It is a C++ Library, http://dlib.net/ (accessed on April 15, 2017).

^5^See the MAGiC App Channel under YouTube, https://www.youtube.com/channel/UC2gvq0OluwpdjVKGSGg-vaQ, and MAGiC App Wiki Page under Github, https://github.com/ulkursln/MAGiC/wiki.

^6^For detailed information, see the website: http://www.fon.hum.uva.nl/praat/.

^7^It is available under https://gist.github.com/ulkursln.

^8^A binary decision tree that can be used while determining the communicative functions and the dimensions is available for annotation of Turkish dialogues, under https://github.com/ulkursln/Dialogue-Act-Annotation.

^9^Please see https://github.com/ulkursln/R-scripts for R scripts.
